# Investigation of the photocatalytic efficiency of tantalum alkoxy carboxylate-derived Ta_2_O_5_ nanoparticles in rhodamine B removal

**DOI:** 10.3762/bjnano.8.65

**Published:** 2017-03-13

**Authors:** Subia Ambreen, Mohammad Danish, Narendra D Pandey, Ashutosh Pandey

**Affiliations:** 1 Department of Chemistry, Motilal Nehru National Institute of Technology, Allahabad, 211004, India

**Keywords:** alkoxy carboxylates, band gap, dynamic light scattering (DLS), rhodamine B, scanning electron microscopy (SEM), tantalum(V) oxide (Ta_2_O_5_) nanoparticles, TEM, X-ray diffraction (XRD)

## Abstract

Ta_2_O_5_ nanoparticles have been synthesized from alkoxy carboxylates of tantalum via the sol–gel route. Tantalum alkoxides were reacted with chlorocarboxylic acids in order to lower the susceptibility hydrolysis. When these modified alkoxy carboxylates were used in the sol–gel synthesis, they yielded Ta_2_O_5_ nanoparticles of better properties than those of the alkoxide-derived Ta_2_O_5_ nanoparticles. These nanoparticles efficiently removed rhodamine B under UV light irradiation.

## Introduction

Nowadays, the purification of water resources has become a major concern. Contamination of water by organic dyes is a threat as these molecules are non-biodegradable and highly toxic. Conventional waste water treatments (to remove solids, organic matter and, sometimes, nutrients from wastewater) such as coarse screening, grit removal, sedimentation and filtration are not very effective in removing organic dyes. Advanced oxidation processes (AOPs) receive a lot of interest in this regard, and photocatalysis by semiconductors is the most extensively investigated AOP. Metal oxide nanoparticles (NPs), for example TiO_2_, ZnO, SnO_2_ and CeO_2_, serve as potential photocatalysts [1–4]. The properties of the metal oxide nanoparticles (surface area, band gap, porosity) determine its photocatalytic activity for the degradation of organic pollutants from water.

Because of properties such as high refractive index and large band gap energy [5–8], Ta_2_O_5_ nanoparticles are an open area for researchers. Tantalum pentoxide is an n-type semiconductor. It absorbs only UV light due to its wide band gap. Nevertheless, band gap modification through various methods has been proven to be successful for the red-shift of the optical absorption of these materials [9–11].

Tantalum(V) alkoxides are important precursors for materials based on tantalum oxide in sol–gel processes. The reactions involved in sol–gel processes can be written as:


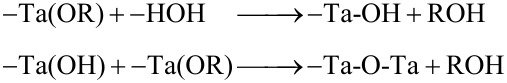


An inorganic network is formed during hydrolysis the ends of which are occupied either by –OH or by –OR groups. Among others, carboxylates are diketonates are employed to control the reactivity and stability towards hydrolysis of tantalum alkoxides [12–14]. Usually, the rate of hydrolysis of metal alkoxides is controlled by increasing the steric hindrance of alkoxy groups, which slows down their replacement by **–**OH groups. This can be achieved by the longer or more branched alkyl groups of **–**OR, or by replacing alkoxo ligands with chelating groups. As a result, a homogenous gel is obtained with a lesser extent of cross-linking.

Bidentate complexing ligands (BL) are usually employed in order to lower the nucleophilicity of the tantalum alkoxides. This is achieved by the incorporation of ligands such as β-diketones, β-ketoesters, carboxylic acids [15–18]. The reaction can be schematized as:


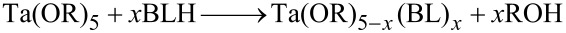


This modification in the parent alkoxide results in several alterations in the new heteroleptic alkoxide. The modified precursors exhibit different physical states, solubilities and reactivities.

In this paper, synthesis and characterization of alkoxy carboxylates of tantalum and tantalum oxide nanomaterials are discussed. Precursors for Ta_2_O_5_ were synthesized from the reactions of Ta(OEt)_5_ (**1**) and Ta(O*n*-Bu)_5_ (**2**) with mono-, di-, and trichloroacetic acid in 1:1 molar ratio in toluene. Ta(OEt)_4_(OOCCH_2_Cl) (**3**), Ta(OEt)_4_(OOCCHCl_2_) (**4**), Ta(OEt)_4_(OOCCCl_3_) (**5**), Ta(O*n*-Bu)_4_(OOCCH_2_Cl) (**6**), Ta(O*n*-Bu)_4_(OOCCHCl_2_) (**7**), Ta(O*n*-Bu)_4_(OOCCCl_3_) (**8**) were used as precursor alkoxy carboxylates. The photocatalytic activity of the nanoparticles was investigated regarding the degradation of rhodamine B (RhB).

## Experimental

All reactions before the sol–gel synthesis were carried out under strict anhydrous conditions by using Schlenk tubes and vacuum line techniques. Ta(OEt)_5_ and Ta(O*n*-Bu)_5_ were purchased from Sigma-Aldrich and used as such for carrying out the reactions. Toluene was dried by standard procedures. ^1^H NMR and ^13^C NMR spectra were recorded in CDCl_3_ and DMSO-*d*_6_ on a Bruker Biospin ARX spectrometer with TMS as internal reference. Ta_2_O_5_ was prepared by sol–gel synthesis through hydrolysis–condensation of tantalum alkoxide and the alkoxy chloroacetate derivatives. X-ray diffraction patterns were recorded on a RIGAKU Smart lab X-ray diffractometer using Cu Kα radiation. The particle size distribution in chloroform dispersion was recorded by a Nanotrac particle analyser. TEM images were taken on a transmission electron microscope JEOL JEM-1011. SEM images were obtained on an EVO MA 15 Zeiss at 15 kV. A Shimadzu UV-2450 UV–vis spectrophotometer was used for recording the absorbance. The surface area was calculated according to the Brunauer–Emmett–Teller (BET) model from N_2_ adsorption in a Micromeritics ASAP 2020 after drying the samples at 200 °C.

### General method for the synthesis of compounds Ta(OR)_4_(OOCR′)

Tantalum alkoxide (1 mmol) was dissolved to dry toluene (20 mL). A solution of the mono/di/trichloroacetic acid (1 mmol) in dry toluene (20 mL) was added dropwise to the stirred solution of alkoxide over a period of 30 min at 25 °C. After stirring at ambient temperature for 10 h the solvent was removed in vacuo to give the compounds.

### General method for the synthesis of Ta_2_O_5_ nanoparticles

The precursor tantalum alkoxide/alkoxy carboxylate (1 mmol) was dissolved in its respective parent alcohol (ethanol or *n*-butanol) (15 mL). The solution was cooled to −84 °C. Double distilled water (18 mmol) was added dropwise under continuous stirring. After the complete addition the solution was allowed to warm up slowly to room temperature leading to the formation of a transparent gel. The obtained gel was left at room temperature for 24 h for aging. It was then dried at 90 °C for 12 h to remove the solvent and other volatile residues to provide a white to off-white powder, which after calcination afforded Ta_2_O_5_ nanoparticles.

### Photocatalytic degradation of rhodamine B over Ta_2_O_5_ nanoparticles

Ta_2_O_5_ nanoparticles (0.8 mg/mL) were dispersed in 50 mL distilled water. The dispersion was charged with RhB (12.5 ppm) after sonication for 20 min at ambient temperature. In order to attain an adsorption–desorption equilibrium the dispersion was stirred in the dark for 45 min prior to UV irradiation. Aliquots of 3 mL were taken after regular intervals of exposure to UV light for recording the absorption of the remaining dye.

The details of the synthesis and physical properties of the compounds **2**–**8** are listed in Table 1.

**Table 1:** Preparative details and physical properties of tantalum alkoxy carboxylates.

reactants (g, mol)	product, yield in g; %	appearance

Ta(OEt)_5_ (0.229, 0.0005) + CH_2_ClCOOH (0.533, 0.0005)	Ta(OEt)_4_(OOCCH_2_Cl), 0.241; 91	viscous yellow liquid
Ta(OEt)_5_ (0.586, 0.0014) + CHCl_2_COOH (0.186, 0.0014)	Ta(OEt)_4_(OOCCHCl_2_), 0.642; 94	low-melting yellow solid
Ta(OEt)_5_ (0.596, 0.0015) + CCl_3_COOH (0.240, 0.0015)	Ta(OEt)_4_(OOCCCl_3_), 0.759; 92	sticky solid
Ta(O*n*-Bu)_5_ (0.388, 0.0007) + CH_2_ClCOOH (0.067, 0.0007)	Ta(O*n*-Bu)_4_(OOCCH_2_Cl), 0.400; 99	white solid
Ta(O*n*-Bu)_5_ (0.523, 0.001) + CHCl_2_COOH (0.123, 0.001)	Ta(O*n*-Bu)_4_(OOCCHCl_2_), 0.523; 98	yellow liquid
Ta(O*n*-Bu)_5_ (0.507, 0.001) + CCl_3_COOH (0.151, 0.001)	Ta(O*n*-Bu)_4_(OOCCCl_3_), 0.580; 92	viscous liquid

The spectroscopic data of compounds **2**–**8** are as follows:

**Ta(OEt)_4_(OOCCH_2_Cl) (3):** ^1^H NMR (25 °C) δ 1.09, 1.21 (t, CH_2_C*H*_3_), 3.50, 4.16 (q, C*H*_2_CH_3_), 4.07 (s, ClC*H*_2_COO); ^13^C NMR (25 °C) δ 18.0 (CH_2_*C*H_3_), 61 (*C*H_2_CH_3_), 84.2 (Cl*C*H_2_COO), 179.5 (ClCH_2_*C*OO).

**Ta(OEt)_4_(OOCCHCl_2_) (4):** ^1^H NMR (25 °C) δ 1.10, 1.19 (t, CH_2_C*H*_3_), 3.56, 4.18 (q, C*H*_2_CH_3_), 5.9 (s, Cl_2_C*H*COO); ^13^C NMR (25 °C) δ 18.1 (CH_2_*C*H_3_), 61.3 (*C*H_2_CH_3_), 85.8 (Cl_2_*C*HCOO), 175 (Cl_2_CH*C*OO).

**Ta(OEt)_4_(OOCCCl_3_) (5):** ^1^H NMR (25 °C) δ 1.12, 1.25 (t, CH_2_C*H*_3_), 3.66, 4.23 (q, C*H*_2_CH_3_); ^13^C NMR (25 °C) δ 18.1 (CH_2_*C*H_3_), 59.8 (*C*H_2_CH_3_), 93 (*C*Cl_3_COO), 189 (CCl_3_*C*OO).

**Ta(O*n*-Bu)_4_(OOCCH_2_Cl) (6):** ^1^H NMR (25 °C) δ 0.92, 0.94 (t, C*H*_3_CH_2_CH_2_CH_2_), 1.38, 1.42 (m, CH_3_C*H*_2_CH_2_CH_2_), 1.56, 1.65 (m, CH_3_CH_2_C*H*_2_CH_2_), 3.65, 4.20 (t, CH_3_CH_2_CH_2_C*H*_2_), 4.05 (s, ClC*H*_2_COO); ^13^C NMR (25 °C) δ 16.2 (*C*H_3_CH_2_CH_2_CH_2_), 22.3 (CH_3_*C*H_2_CH_2_CH_2_), 43 (CH_3_CH_2_*C*H_2_CH_2_), 69 (C*H*_3_CH_2_CH_2_CH_2_), 85 (Cl*C*H_2_COO), 179 (ClCH_2_*C*OO).

**Ta(O*n*-Bu)_4_(OOCCHCl_2_) (7):** ^1^H NMR (25 °C) δ 0.94 (t, C*H*_3_CH_2_CH_2_CH_2_), 1.48 (m, CH_3_C*H*_2_CH_2_CH_2_), 1.64 (m, CH_3_CH_2_C*H*_2_CH_2_), 3.62, 4.13 (t, CH_3_CH_2_CH_2_C*H*_2_), 6.08 (s, Cl_2_C*H*COO); ^13^C NMR (25 °C) δ 16.2 (*C*H_3_CH_2_CH_2_CH_2_), 25 (CH_3_*C*H_2_CH_2_CH_2_), 44.6 (CH_3_CH_2_*C*H_2_CH_2_), 65.4 (C*H*_3_CH_2_CH_2_CH_2_), 87 (Cl_2_*C*HCOO), 182 (Cl_2_CH*C*OO).

**Ta(O*n*-Bu)_4_(OOCCCl_3_) (8):** ^1^H NMR (25 °C) δ 0.93, 0.95 (t, C*H*_3_CH_2_CH_2_CH_2_), 1.40 (m, CH_3_C*H*_2_CH_2_CH_2_), 1.62 (m, CH_3_CH_2_C*H*_2_CH_2_), 3.65, 4.28 (t, CH_3_CH_2_CH_2_C*H*_2_); ^13^C NMR (25 °C) δ 16.5 (*C*H_3_CH_2_CH_2_CH_2_), 22 (CH_3_*C*H_2_CH_2_CH_2_), 48.5 (CH_3_CH_2_*C*H_2_CH_2_), 69 (C*H*_3_CH_2_CH_2_CH_2_), 95 (Cl_3_*C*COO), 190 (Cl_3_C*C*OO).

## Results and Discussion

Tantalum(V) alkoxides were reacted with either mono-, di- or trichloroacetic acids to prepare new heteroleptic alkoxides. In order to synthesize mono-substituted alkoxides, tantalum alkoxides were reacted with a stoichiometric amount (1:1) of chlorocarboxylic acids in dry toluene at room temperature. After the completion of reaction, reaction mixture was concentrated and stored in inert atmosphere. The schematic representations of the reactions are as follows:


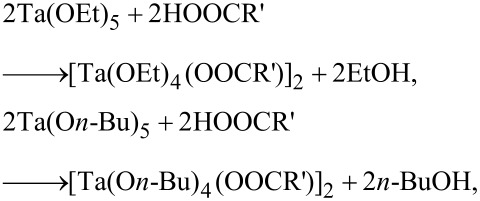


where R′ = CH_2_Cl, CHCl_2_, CCl_3_.

The ^1^H NMR spectrum of compound **3** (Figure S1, Supporting Information File 1) comprises of two triplets at δ 1.09 and 1.21, which are attributed to the methyl protons of ethoxy ligands. The methylene protons of the ethoxy group are represented by the pair of quartets at δ 3.50 and 4.16. The signal for protons of monochloroacetate is shown by the singlet at δ 4.07. We may say that the two sets for ethoxy protons correspond to the terminal and bridging ethoxy groups.

Further, in the ^1^H NMR spectrum of compound **6** (Figure S2, Supporting Information File 1) triplets at δ 0.92 and 0.94 are allotted to the methyl protons (C*H*_3_CH_2_CH_2_CH_2_). The methylene protons at C3 position (CH_3_C*H*_2_CH_2_CH_2_) are represented by two multiplets at δ 1.38 and 1.42. The two multiplets at δ 1.56 and 1.65 are designated to CH_3_CH_2_C*H*_2_CH_2_ methylene protons. The peaks at δ 3.65 and 4.20 stand for CH_3_CH_2_CH_2_C*H*_2_ methylene protons. It is assumed that there are also terminal and bridging alkoxy groups. The singlet at δ 4.05 is assigned to the protons of chloroacetate ligand. Likewise information is gained from the NMR spectra of other modified tantalum ethoxides and butoxides.

Based on the obtained data it may be said that there are two types of alkoxy ligands in the heteroleptic tantalum alkoxide derivatives: terminal and bridging alkoxy ligands. It may be proposed that these compounds exist in dimeric forms bridged by two bridging alkoxy groups and two bridging bidentate carboxylates (Figure S3, Supporting Information File 1). Each metal is ligated to terminal alkoxy groups. It is noticeable here that the dimeric nature of parent alkoxides is sustained in the heteroleptic tantalum alkoxides. The dimeric structure of modified tantalum alkoxides are also established by other researchers. Researchers have isolated and confirmed the dimeric nature of heteroleptic alkoxides by single-crystal X-ray diffraction.

Ta_2_O_5_ nanoparticles were synthesized by the sol–gel method. Tantalum(V) alkoxides and the heteroleptic chlorocarboxylato derivatives of tantalum alkoxides were subjected to hydrolysis and condensation reactions. The obtained particles were dried earlier to calcination to remove adsorbed water, impurities and volatilities. Ta_2_O_5_ nanoparticles obtained from Ta(OR)_5_ and Ta(OR)_4_(OOCR′) (R = CH_2_CH_3_/CH(CH_3_)_2_/CH_2_CH_2_CH_2_CH_3_, R′ = CH_2_Cl/CHCl_2_/CCl_3_) displayed different properties. The chloroacetate (OOCR′) group plays a crucial role in deciding the final quality of tantalum oxide. The chelating OOCR′ ligand is hydrolyzed more slowly than the alkoxo group. As a consequence the gelation time is longer for the heteroleptic tantalum alkoxides. The various properties of prepared nanoparticles are discussed in the following.

### XRD analysis

**Figure 1:**
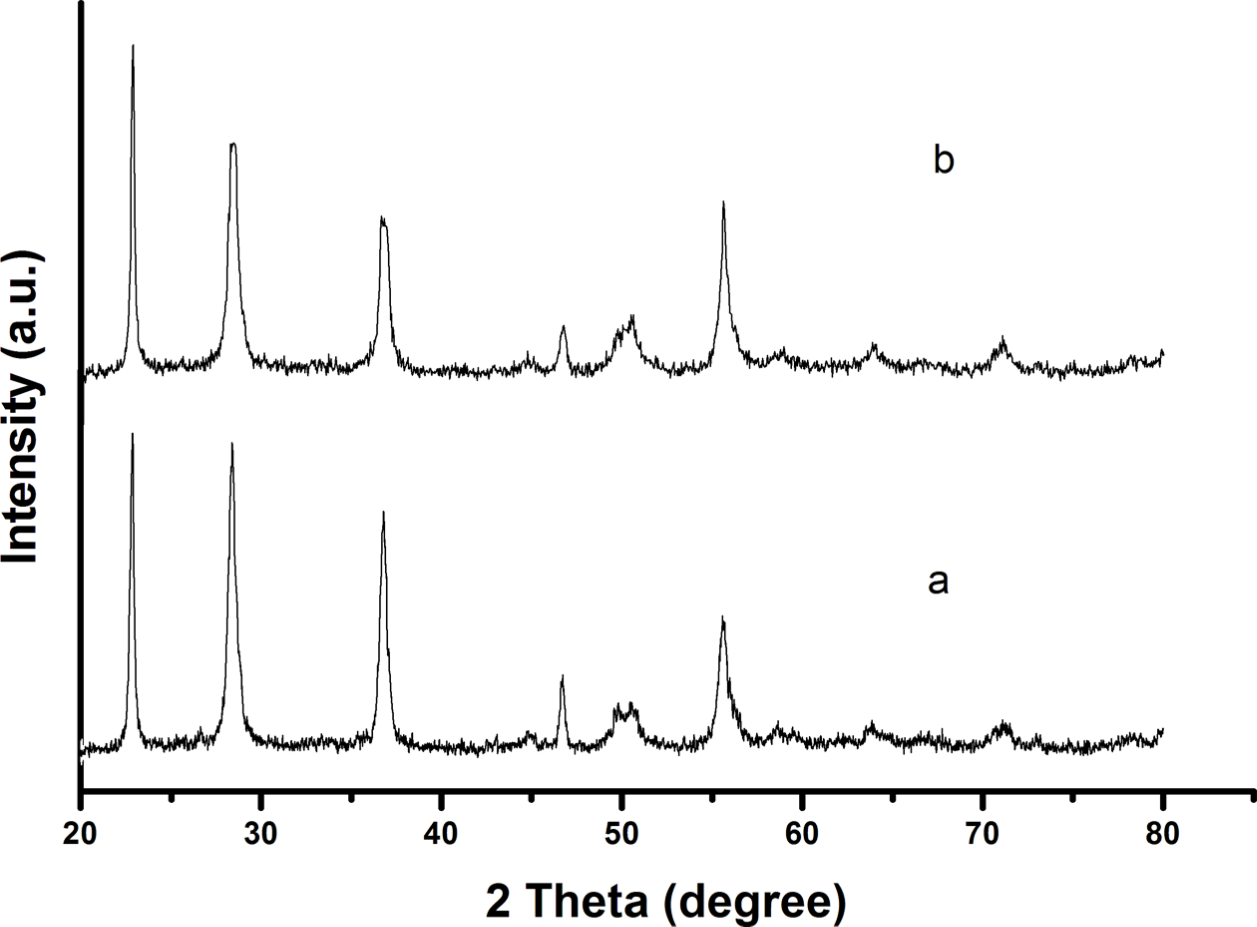
XRD patterns of Ta_2_O_5_ nanoparticles obtained from (a) Ta(OEt)_4_(OOCCHCl_2_) and (b) Ta(O*n*-Bu)_4_(OOCCHCl_2_) calcined at 750 °C for 4 h.

The crystalline phase progression of tantalum pentoxide nanoparticles has been examined using XRD measurements. Amorphous behavior was observed for the as-synthesized Ta_2_O_5_ nanoparticles. XRD peaks intensify significantly and become much sharper with the rise in calcination temperature indicating that the crystallinity of Ta_2_O_5_ nanoparticles increases. As the calcination temperature is elevated to 750 °C, several intense and sharp diffraction peaks appeared. XRD patterns of Ta_2_O_5_ samples prepared from different precursor alkoxides and calcined at 750 °C for 4 h are shown in Figure 1. The nanoparticles synthesized from tantalum ethoxide and tantalum *n*-butoxide are less crystalline than those obtained from their chloroacetato derivatives, Ta(OEt)_4_(OOCR) and Ta(O*n*-Bu)_4_(OOCR′) (where R′ = CH_2_Cl, CHCl_2_ and CCl_3_). The peaks at 2θ values 22.88, 28.77, 36.66, 46.64 and 55.60° correspond to (001), (100), (101), (002) and (102) crystallographic planes of the orthorhombic phase of Ta_2_O_5_ [19]. The reason behind this observation can be attributed to the fact that the cross-linking of the gel is reduced in heteroleptic carboxylato tantalum alkoxides compared to the homoleptic alkoxides. Also, steric hindrance by the chloroacetate ligands might have directed the pathway of gel networking in the precursor alkoxides.

The average particle size of Ta_2_O_5_ nanoparticles is calculated with the help of the Scherrer equation:


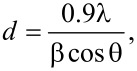


where λ = X-ray wavelength, β = FWHM (full width at half maximum) and θ = angle. The average grain sizes of Ta_2_O_5_ nanoparticles synthesized from all precursors are found to be in range of 12–32 nm (Table 2). Ta_2_O_5_ nanoparticles synthesized from Ta(OEt)_5_ and Ta(OEt)_4_(OOCCHCl_2_) exhibit particle sizes of 15 and 21 nm and are smaller than those obtained from Ta(O*n*-Bu)_5_ (17 nm) and Ta(O*n*-Bu)_4_(OOCCHCl_2_) (23 nm). It is well known that branching and increasing the length of alkyl group of alkoxy ligands in precursor alkoxide increases the gelation time and therefore, results in more crystalline products with larger particle sizes [20].

**Table 2:** Properties of Ta_2_O_5_ nanoparticles.

Ta_2_O_5_ precursor	average particle size (nm)	TOPO-coated particle size (nm)	band gap (eV)	surface area (m^2^·g^−1^) / pore volume (cm^3^·g^−1^)

Ta(OEt)_5_	15	155	3.5	—
Ta(OEt)_4_(OOCCH_2_Cl)	18	—	3.4	48/0.25
Ta(OEt)_4_(OOCCHCl_2_)	21	147	3.25	45/0.29
Ta(OEt)_4_(OOCCHCl_2_)_2_	—	—	—	—
Ta(O*n*-Bu)_5_	17	159	3.5	61/0.13
Ta(O*n-*Bu)_4_(OOCCHCl_2_)	23	141	3.15	42/0.32

### TEM and SEM analysis

**Figure 2:**
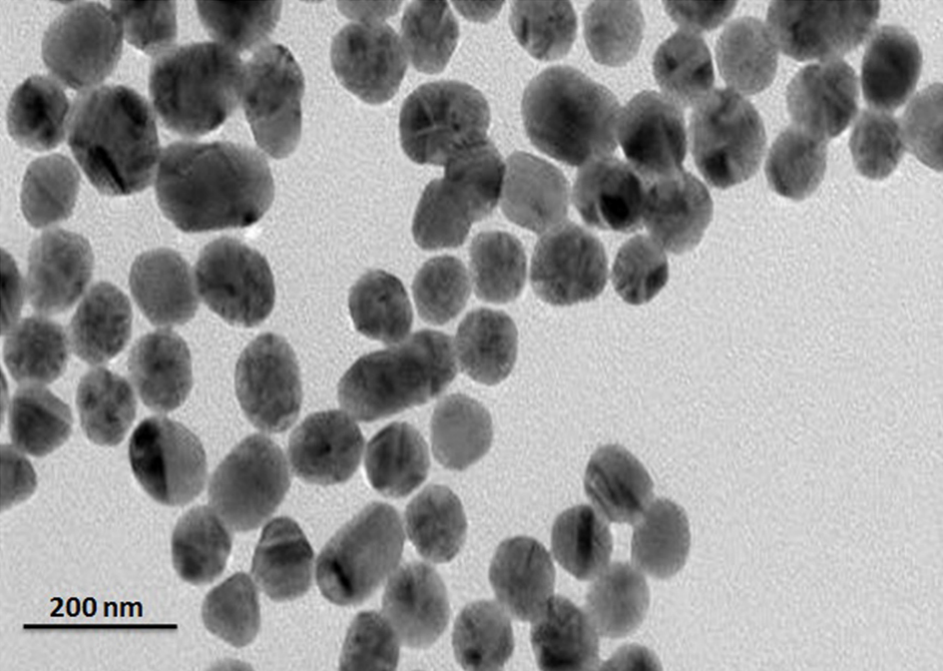
TEM image of Ta_2_O_5_ nanoparticles obtained from Ta(OEt)_4_(OOCCH_2_Cl).

**Figure 3:**
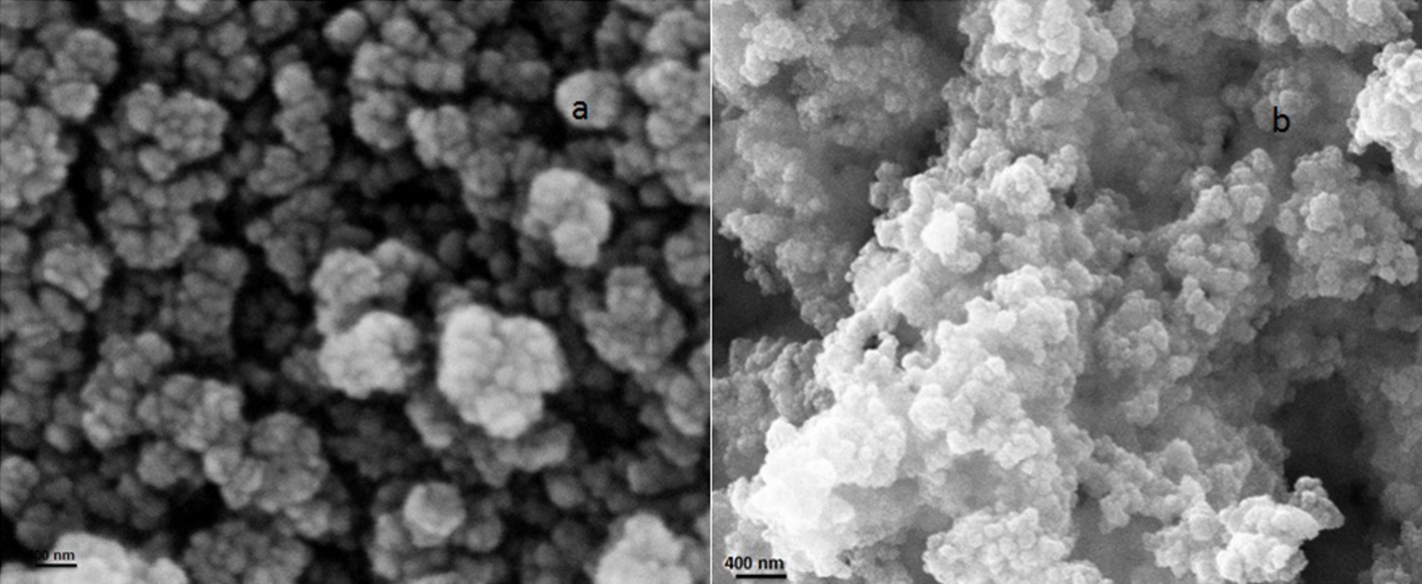
SEM images of Ta_2_O_5_ nanoparticles obtained from (a) Ta(OEt)_4_(OOCCHCl_2_) and (b) Ta(O*n*-Bu)_4_(OOCCHCl_2_).

To study the morphology and texture of the synthesized Ta_2_O_5_ nanoparticles TEM and SEM were carried out. A TEM image of Ta(OEt)_4_(OOCCH_2_Cl)-derived Ta_2_O_5_ nanoparticles shows that the particles are spherical with an average diameter of 40 nm (Figure 2). SEM micrographs of calcined Ta_2_O_5_ nanoparticles are shown in Figure 3. Ta_2_O_5_ particles derived from different tantalum alkoxides show different morphologies, particle sizes and distributions. Apparently, the particles are roughly spherical and agglomerated to some level. The agglomeration is higher in case of Ta_2_O_5_ synthesized from homoleptic alkoxides, Ta(OEt)_5_ and Ta(O*n*-Bu)_5_, than in the chloroacetate-modified tantalum alkoxides. Ta_2_O_5_ nanoparticles synthesized from dichloroacetato tantalum ethoxide are round in shape and their diameter is 150 nm. The diameters of all prepared Ta_2_O_5_ nanoparticles samples are found to be in the range of 120–170 nm. The oxide nanoparticles are moderately evenly dispersed in all cases.

### DLS measurements

**Figure 4:**
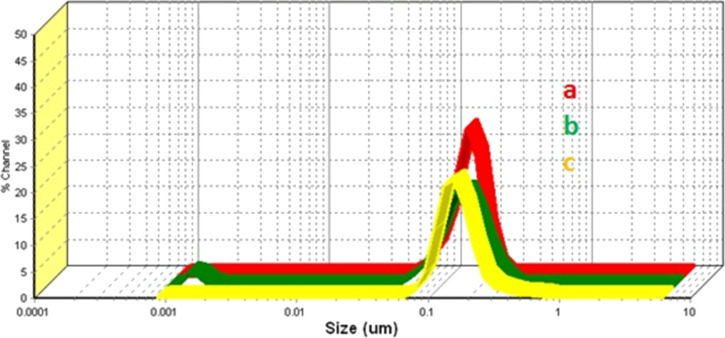
Particle sizes and distributions of Ta_2_O_5_ synthesized from (a) Ta(O*n*-Bu)_4_(OOCCH_2_Cl), (b) Ta(O*n*-Bu)_4_(OOCCHCl_2_) and (c) Ta(O*n*-Bu)_4_(OOCCCl_3_).

To study the properties of nanoparticles in dispersion dynamic light scattering (DLS) experiments were carried out. Stable dispersions of Ta_2_O_5_ nanoparticles in chloroform were prepared by using the surfactant trioctylphosphine oxide (TOPO). TOPO molecules provide stability and uniformity to the nanoparticles in chloroform. Table 2 shows the obtained particle sizes and distributions of Ta_2_O_5_ in chloroform dispersions. It can be seen that the TOPO-coated Ta_2_O_5_ particles derived from different precursors exhibit different particle sizes and distributions. Figure 4 shows the particle size and distribution of TOPO-coated Ta_2_O_5_ obtained from different precursors. Ta(O*n*-Bu)_4_(OOCCCl_3_)-derived nanoparticles were found to display agglomeration in the suspension. The distribution of particles is more uniform in the nanoparticles derived from carboxylato alkoxides than in those derived from alkoxides.

### UV–vis spectroscopy

**Figure 5:**
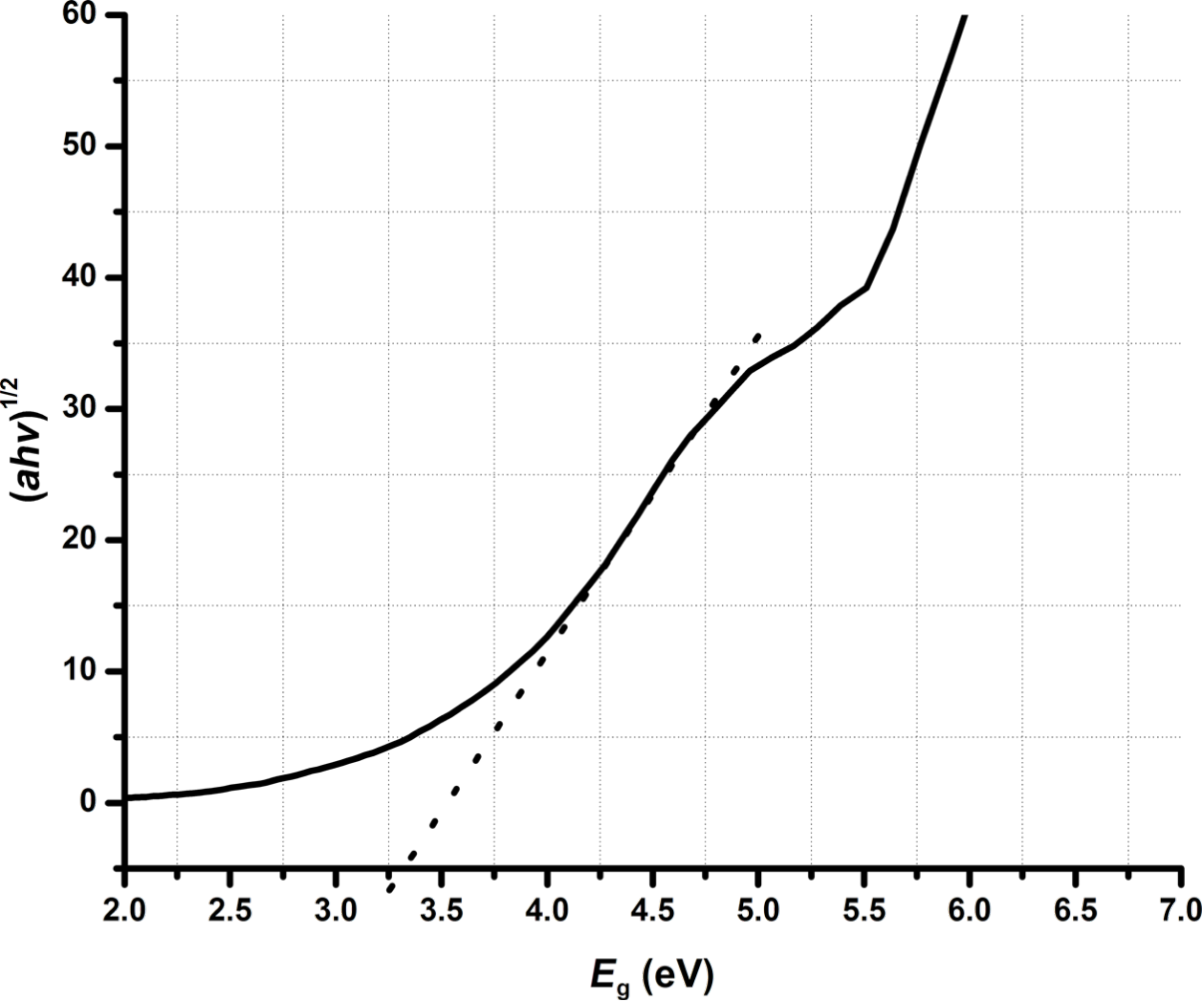
Solid-state diffuse reflectance UV–vis spectrum and extrapolation of the band gap energy of Ta_2_O_5_ nanoparticles prepared from Ta(OEt)_5_.

The optical properties of synthesized Ta_2_O_5_ nanoparticles were studied by UV–vis diffuse reflectance spectroscopy (DRS). The synthesized Ta_2_O_5_ nanoparticles display a characteristic absorbance onset at ca. 360 nm (Figure 5). The deviation in the absorbance of nanoparticles may be attributed to the disparity in crystallite size and surface morphologies. The band gap is calculated from the Tauc plot [21]. Band gap energy and particle size are in inversely proportional. The band gap energies of Ta_2_O_5_ nanoparticles produced from Ta(OEt)_4_(OOCCHCl_2_) and Ta(O*n*-Bu)_4_(OOCCHCl_2_) are found to be 3.25 eV and 3.15 eV and smaller than earlier estimated values [22–25]. The band gap energies of Ta_2_O_5_ nanoparticles produced from different alkoxy carboxylates are found to be about 3.5 eV.

### BET analysis

N_2_ adsorption and the Brunauer–Emmett–Teller (BET) theory were used to calculate the surface areas of the calcined Ta_2_O_5_ nanoparticles. The powders were dried at higher temperatures prior to analysis in order to remove moisture. The surface areas of various nanoparticles are given in Table 2. Smaller particles exhibit larger surface areas.

The difference in various properties of Ta_2_O_5_ nanoparticles synthesized from alkoxides and alkoxy carboxylates may be attributed to the fact that the evolution of the sol–gel process in both the cases is different. The chelating carboxylates substitute the alkoxy group of the metal alkoxide to enhance the steric effects and create hindrance for the nucleophilic attack of water molecules on the alkoxide. This slows down the hydrolysis effectively, which in turn influences the structure of the gel [26].

### Degradation of rhodamine B

The photocatalytic degradation of rhodamine B by Ta_2_O_5_ nanoparticles under UV irradiation has been studied. The prepared nanoparticles decomposed the dye by a series of photochemical reactions. An electron–hole pair is generated when the semiconductor absorbs photons. These electron–hole pairs migrate to the surface and react with adsorbed water molecules or hydroxide ions to produce hydroxyl radicals. These hydroxyl radicals decompose the dye molecule into CO_2_ and water. The concentration of remaining dye in the solution is measured by using UV–vIS spectroscopy. The formula employed for the calculation of the remaining dye is as follows [27–29]:


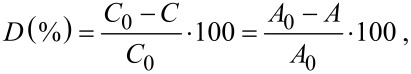


where *C* and *C*_0_ are the measured and initial concentrations, and *A*_o_ and *A* are the initial and the measured absorption intensities of the rhodamine B.

**Figure 6:**
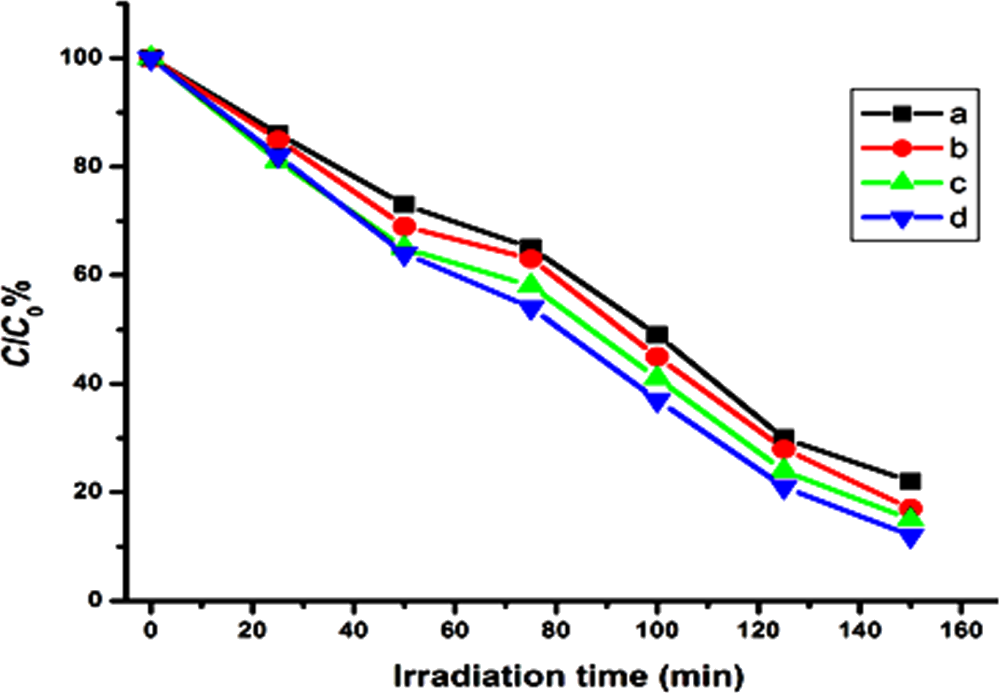
Degradation of RhB by Ta_2_O_5_ nanoparticles synthesized from (a) Ta(OEt)_5_, (b) Ta(OEt)_4_(OOCCH_2_Cl), (c) Ta(OEt)_4_(OOCCHCl_2_) and (d) Ta(OEt)_4_(OOCCCl_3_).

**Figure 7:**
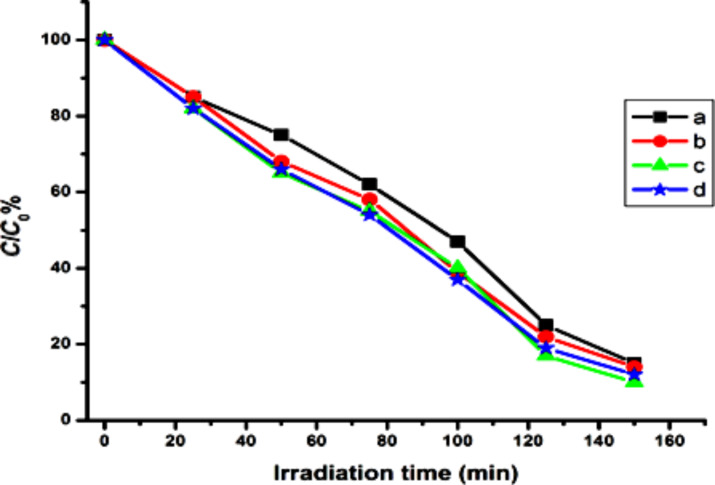
Degradation of RhB by Ta_2_O_5_ nanoparticles synthesized from (a) Ta(O*n*-Bu)_4_, (b) Ta(O*n*-Bu)_4_(OOCCH_2_Cl), (c) Ta(O*n*-Bu)_4_(OOCCHCl_2_) and (d) Ta(O*n*-Bu)_4_(OOCCCl_3_).

It is seen that the Ta_2_O_5_ nanoparticles removed the dye color effectively (Figure 6, Figure 7). Since the chloroacetate ligands employed for the substitution of alkoxy groups are similar in nature there is a slight difference in the properties and photocatalytic efficiency of the final nanoparticles. The variation in particle size, band gap energy and surface area are the reasons for the difference of catalytic performances of nanoparticles. The nanoparticles were recycled from the reactions (after centrifugation, washing and drying) and reused, however, the efficiency of the reused catalysts was decreased by more than 50%.

**Figure 8:**
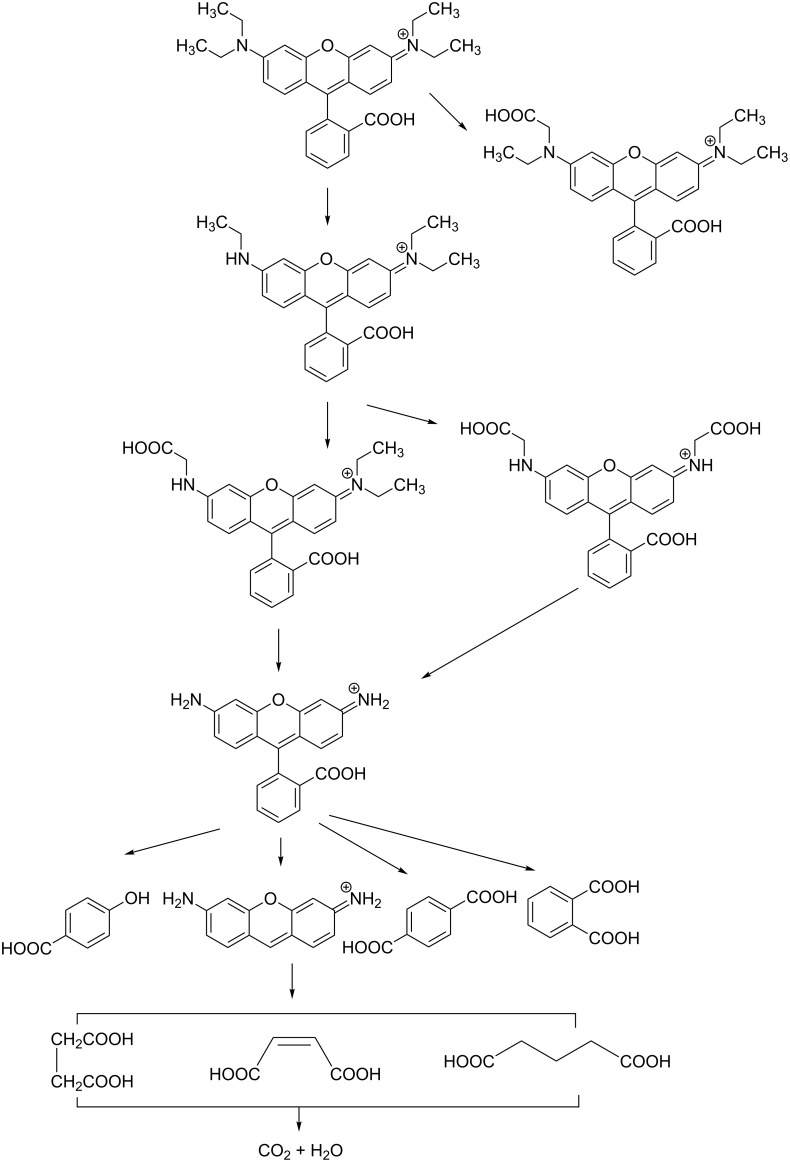
Possible intermediates of rhodamine B during photocatalytic degradation process.

The mechanism of photocatalytic degradation of RhB is supposed to involve a series of carboxylation and de-ethylation reactions until the dye is decomposed into CO_2_ and H_2_O. The proposed intermediates in degradation process are shown in Figure 8 [30–31].

The degradation of RhB in absence of the catalyst was neglible (2–4%). The photocatalytic degradation of RhB was modeled with the Langmuir–Hinshelwood mechanism, which is most commonly used to explain the kinetics of heterogeneous photocatalytic reaction [30]. It is expressed as follows:


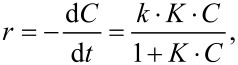


where *r* is reaction rate, *k* is the reaction rate constant, *K* is the adsorption coefficient, *t* is the time and *C* is the concentration of the reactant. If *C* is small then the reaction can be described to be of pseudo-first order:


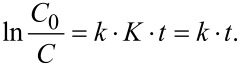


**Figure 9:**
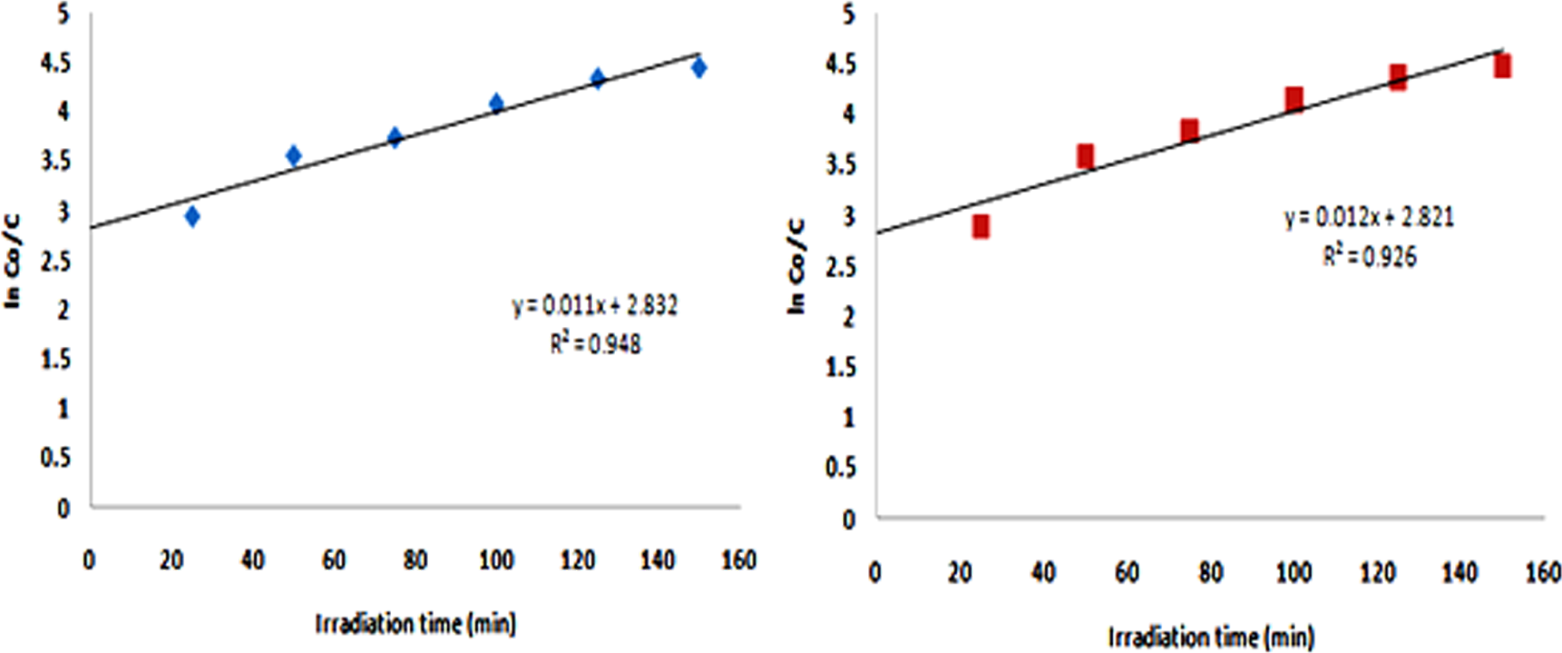
Graphical determination of the reaction rate of the photocatalytic degradation of RhB by Ta_2_O_5_ nanoparticles derived from (a) Ta(OEt)_4_(OOCCHCl_2_) and (b) Ta(OEt)_4_(OOCCCl_3_).

The plots of ln(*C*_0_/*C*) as a function of the time give straight lines in which the slope represents *k* (Figure 9). The value of *k* for different reactions is listed in Table 3.

**Table 3:** Rate constants of photocatalytic degradation reactions of RhB.

metal oxide NPs	precursor alkoxide	*k* (min^−1^)

Ta_2_O_5_	Ta(OEt)_4_(OOCCHCl_2_)	0.011
Ta_2_O_5_	Ta(OEt)_4_(OOCCCl_3_)	0.012

## Conclusion

Heteroleptic tantalum alkoxides were successfully synthesized by reactions of tantalum ethoxide and tantalum *n*-butoxide with mono-/di-/trichloroacetic acid in inert atmosphere. The obtained products are pure and exhibit different physicochemical properties than their parent alkoxides. The modified compounds are also moisture sensitive. However, the sensitivity is expected to be lower than that of the homoleptic alkoxides. NMR spectra reveal the presence of two types of alkoxy groups in the compounds: terminal and bridging. The spectroscopic details of these compounds explain that all these are dimeric in nature, i.e., the geometry of parent alkoxides is maintained. The replacement of alkoxy groups by chloroacetate ligands in Ta(OR)_5_ (OR = OEt/O*n*-Bu) slows down the hydrolysis during the sol–gel process. The bidentate carboxylate groups are difficult to hydrolyze and therefore, alkoxy groups are preferentially hydrolyzed. The process of gelation is enhanced and oligomerization is controlled in these precursors.

Ta_2_O_5_ nanoparticles were synthesized using the sol–gel method from alkoxy chloroacetates of tantalum. Ta_2_O_5_ nanoparticles are found to be in orthorhombic phase. The particle sizes, as calculated with the Scherrer equation, are in the range of 15–28 nm. The samples attained good crystallinity after calcination at 750 °C. SEM micrographs show that the synthesized samples are almost spherical and agglomerate to some extent.

The photodegradation of rhodamine B by the semiconductor photocatalyst has also been investigated. Electron–hole pairs are generated during exposure to the UV radiation. These charge carriers produce radicals for the decomposition of the dye. The synthesized Ta_2_O_5_ nanoparticles removed rhodamine B efficiently under UV irradiation. It is observed that the photocatalytic efficacy of nanoparticles largely depends on the band gap energy and the surface area. Low band gap energies and high surface areas promote the degradation of the dye. The tantalum pentoxide nanoparticles are proficient in the rhodamine B degradation under UV irradiation.

## Supporting Information

^1^H NMR spectra of modified tantalum alkoxides are shown in Supporting Information.

File 1 Additional experimental data.
