# Supplementary material for: Investigation of the photocatalytic efficiency of tantalum alkoxy carboxylate-derived Ta2O5 nanoparticles in rhodamine B removal
Source: Beilstein J Nanotechnol. 2017 Mar 13;8:604–13. doi: 10.3762/bjnano.8.65 (PMC5372746; doi:10.3762/bjnano.8.65)
Supplement: File 1 — Additional experimental data. [file Beilstein_J_Nanotechnol-08-604-s001.pdf]

Supporting Information

for

Investigation of the photocatalytic efficiency of tantalum alkoxy carboxylate-derived Ta_2O_5 nanoparticles in rhodamine B removal

Subia Ambreen, Mohammad Danish, Narendra D. Pandey and Ashutosh Pandey*,§

Address: Department of Chemistry, Motilal Nehru National Institute of Technology, Allahabad, 211004, India

Email: Ashutosh Pandey * - apandey@mnnit.ac.in

* Corresponding author

§ Phone: +91 9235507541

Additional experimental data

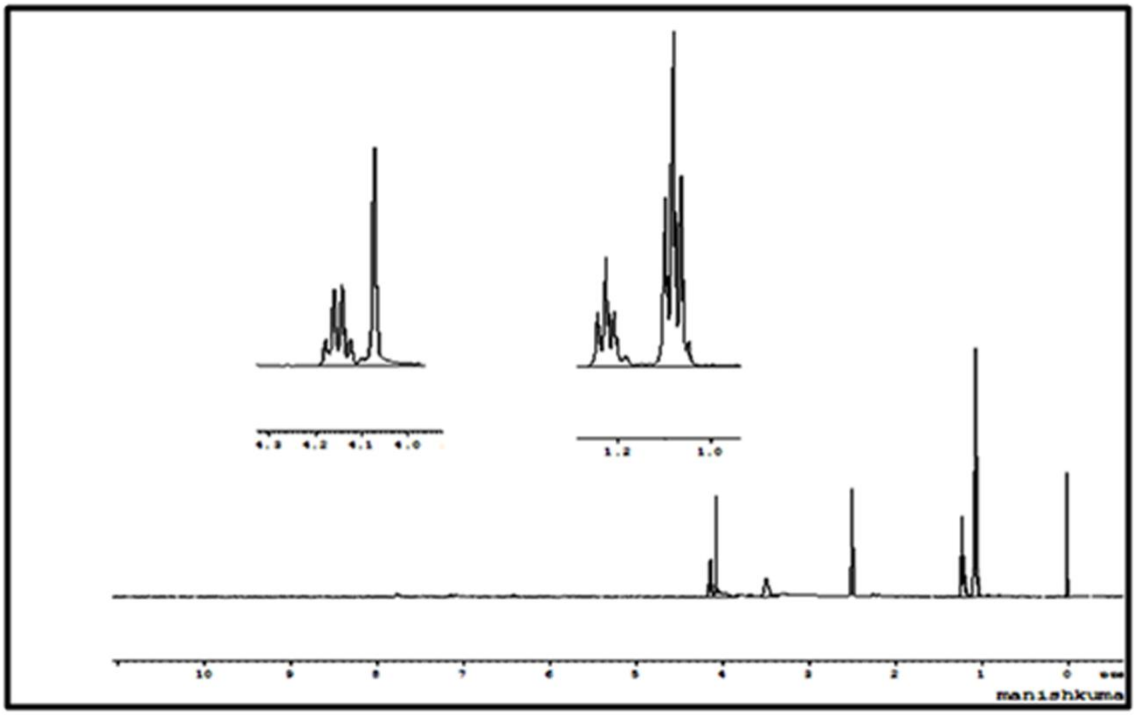

Figure S1: ^{1}H NMR spectrum of $Ta(OEt)_4(OOCCH_2Cl)$.

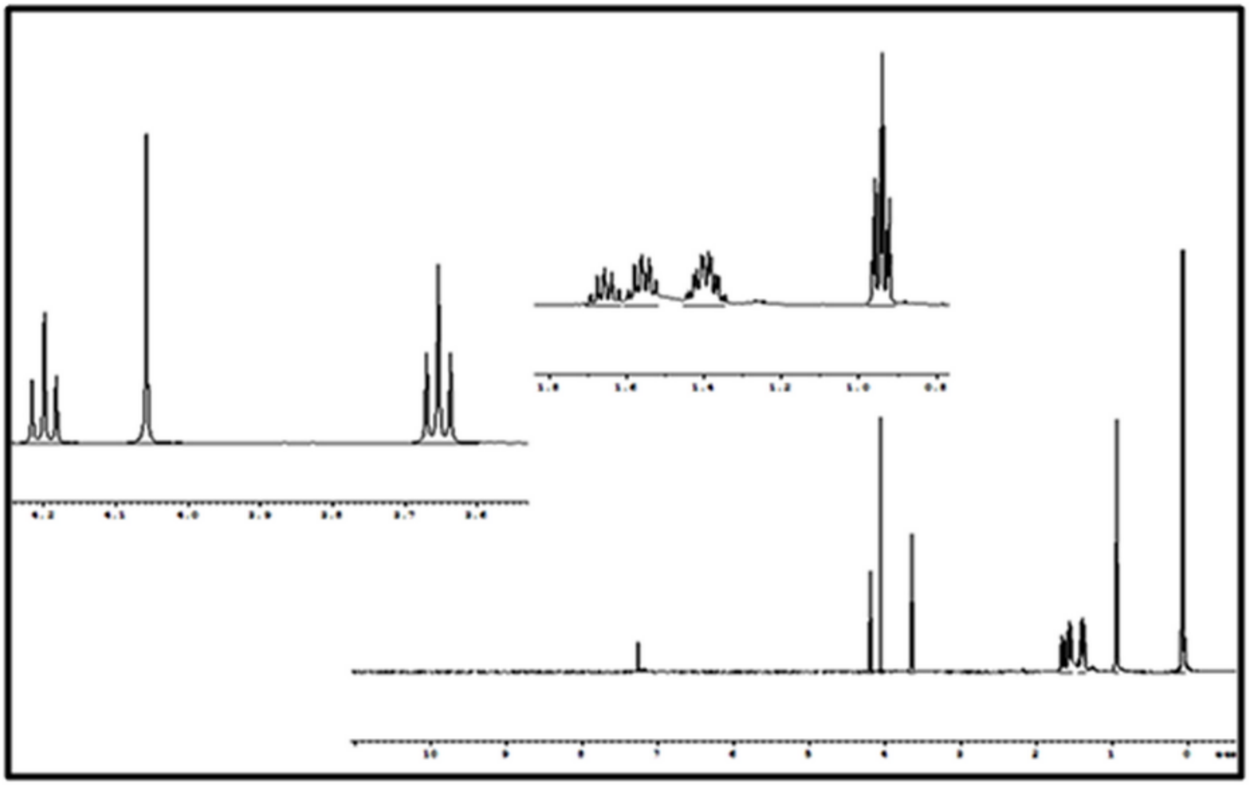

Figure S2: ^{1}H NMR spectrum of Ta(O*n*-Bu)$_4$(OOCCH$_2$Cl).

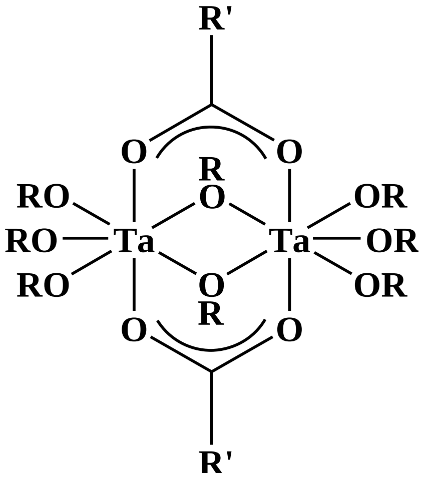

Figure S3: Proposed structure of Ta(OR)$_4$(OOCR′).
